# Measurement of the IgG Avidity Index in the Diagnosis of Clinical Toxocariasis Patients

**DOI:** 10.3390/pathogens10091086

**Published:** 2021-08-26

**Authors:** Estelle Menu, Lora Kopec, Léa Luciani, Sophie Legrand, Coralie L’Ollivier

**Affiliations:** 1 Laboratoire de Parasitologie-Mycologie, Institut Hospitalo-Universitaire Méditerranée Infection, 13385 Marseille, France; estelle.menu@ap-hm.fr (E.M.); lora.kopec@gmail.com (L.K.); sophie.legrand@ap-hm.fr (S.L.); 2 VITROME: Vecteurs-Infections Tropicales et Méditerranéennes, Service de Santé des Armées, Assistance Publique-Hôpitaux de Marseille, Institut de Recherche pour le Développement, Aix Marseille Université, 13385 Marseille, France; 3 Unité des Virus Emergents (UVE), Aix Marseille Université, IRD 190, INSERM 1207, 13385 Marseille, France; lea.luciani@ap-hm.fr

**Keywords:** avidity, *Toxocara* spp., toxocariasis, ELISA, immunoblotting

## Abstract

*Toxocara* spp. are parasitic nematodes responsible for human toxocariasis, a common zoonotic helminth infection. The five main features of human toxocariasis are the classical ocular toxocariasis and visceral larva migrans syndrome, followed by covert toxocariasis, common toxocariasis and neurotoxocariasis. The diagnosis of toxocariasis is feasible by considering clinical symptoms, anamnestic history and serology laboratory results; however, serological criteria cannot be used to distinguish active *Toxocara* infection from past exposure, which is an area of much discussion in clinical practice. In this context, we developed avidity tests (ELISA and immunoblotting) and evaluated their clinical usefulness in distinguishing past from active toxocariasis. Our study involved 46 patients divided into two groups: “active toxocariasis” (n = 14) and “chronic toxocariasis” (n = 32). According to the avidity indices obtained for both the chronic and active toxocariasis groups, we proposed two thresholds: first, an AI lower than 32% supports an active infection; secondly, a threshold above 42% can exclude an active infection. In order to use this assay in routine clinical practice, however, is still requires standardisation with regards to the method and threshold values, which can be established through studies involving larger populations.

## 1. Introduction

*Toxocara canis* and *Toxocara cati* are parasitic nematodes (roundworms) responsible for human toxocariasis (larval toxocariasis), a common zoonotic helminth infection. The seroprevalence of human toxocariasis varies widely across the world, with disparity between rural and urban areas, although it is higher in non-affluent populations [1]. The seroprevalence estimates range from 5 to 15% in the United States, reaching up to 80% in children in parts of Nigeria [2,3]. This neglected disease usually occurs in children because they often play in areas containing contaminated soil. In fact, stray and domiciliated dogs and cats play an important role in the transmission of *Toxocara* spp. by providing environmental contamination opportunities, which perpetuates the spreading of the infection among human populations [4]. Additionally, *Toxocara* spp. is commonly found in the intestines of canine or feline hosts. Humans are paratenic hosts who become infected by ingesting infective eggs in contaminated soil, in raw vegetables or other foods, and possibly from contact with dog or cat hair [5]. After ingestion, the eggs release larvae in the intestine, which migrate throughout the soft tissues of the body (liver, heart, lungs, brain, muscle, eyes). While the larvae do not undergo any further development in these sites, they can cause several local reactions that are the basis of toxocariasis. The five main features of human toxocariasis are classic ocular toxocariasis (OT) and visceral larva migrans (VLM) syndrome, followed by toxocariasis, common toxocariasis and neurotoxocariasis. Common toxocariasis is characterised by a normal or mildly elevated blood eosinophil count and multiple minor symptoms, such as weakness, pruritis, rash, breathing difficulties and abdominal pain. Covert toxocariasis is characterised by inapparent or mild symptoms with or without eosinophilia [6]. The diagnosis of toxocariasis is feasible by considering clinical symptoms, anamnestic history and laboratory results. The sampling of tissue biopsies or fluid samples is invasive and can be impractical, so toxocariasis diagnosis relies on the use of serological techniques. The indirect enzyme-linked immunosorbent assay (ELISA) for antibody detection is currently the most common diagnostic method, which uses standardised antigens (i.e., *T. canis* excretory or secretory (TES)). Initial serological findings should be confirmed by immunoblotting to avoid false-positive results caused by cross-reactivity with other infective agents; however, serological criteria are unable to distinguish an active *Toxocara* infection from past exposure, which is an area of much discussion in clinical practice. The IgG avidity index represents the strength of the bonds between antigens and the corresponding IgG antibodies. In immunocompetent patients, the measurement of the lgG avidity index (AI) is used as an additional test to help date infections. The use of this test is based on the fact that after a primary infection, the antibody response matures from low avidity to high avidity over a period of several weeks to several months. Measurement of the avidity index is very useful for maternofoetal infections, such as toxoplasmosis and cytomegalovirus (CMV) infections [7,8]. AI values can also help in differentiating between past and current toxocariasis infections. Recently, some studies have suggested determining the avidity using the immunoblot technique to discriminate between antigens related to high-avidity antibodies from those related to low-avidity antibodies in strongyloidiasis and Q fever infections [9,10].

Based on these established practices regarding the use of urea for avidity tests, we developed avidity tests (ELISA and immunoblotting) and evaluated their clinical usefulness to distinguish past from active toxocariasis.

## 2. Results

**Figure 1:**
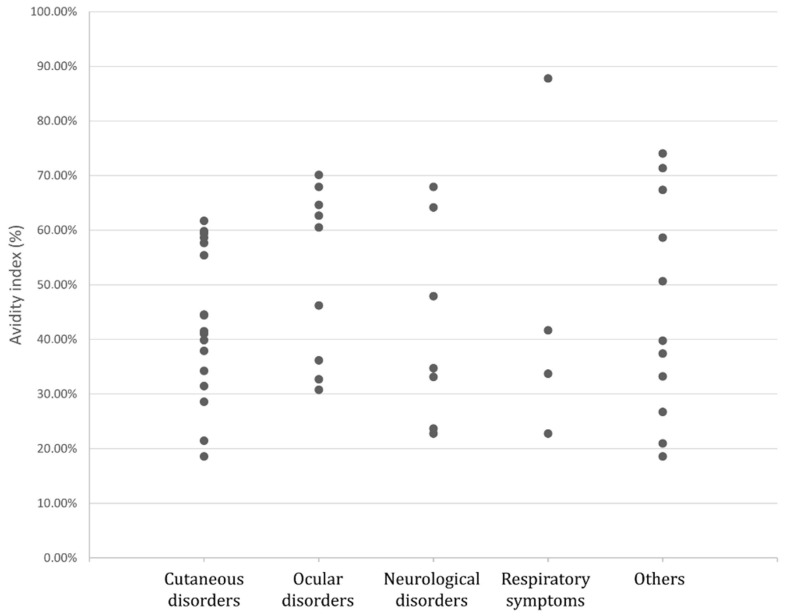
Avidity index results according to clinical symptoms.

**Figure 2:**
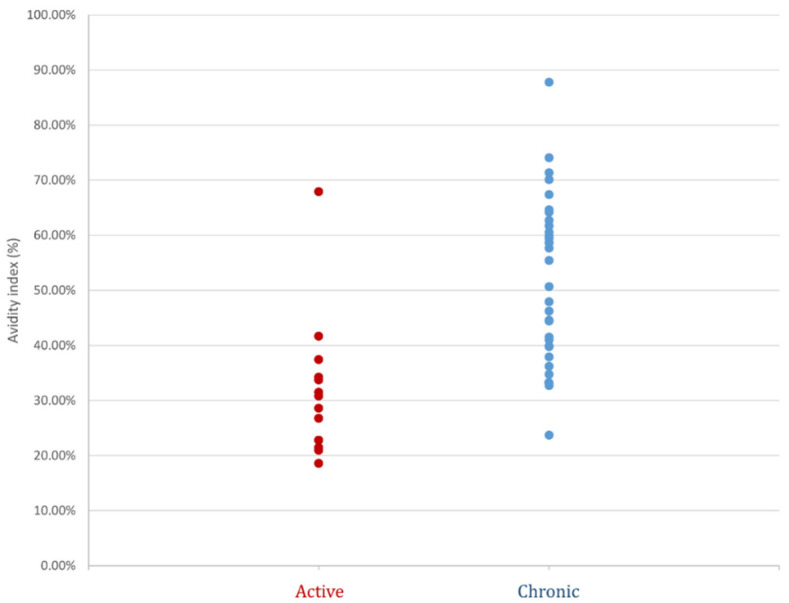
Avidity index results for the 46 patients from the active (red circle) and chronic (blue circle) groups.

Among the 46 patients, when considering clinical presentation overall, cutaneous disorders were the most frequent (37% of patients) (Figure 1). Other clinical manifestations in order of frequency were ocular disorders (20%), neurological disorders (15%) and respiratory symptoms (9%). Other symptoms included asthenia, abdominal pain and swollen lymph nodes (Figure 2). Patients with eosinophilia (eosinophil higher than 0.50 Giga/l) represented 37% of the entire group and 64% of the active group.

AI was determined for the 46 sera samples. The distribution of the AI according to the two groups enabled the creation of the 32% threshold, below which the infection was considered active, as well as the 42% threshold, above which the infection was considered chronic (Figure 2). The <32% threshold was used to confirm active infection with 96.9% specificity, 64.3% sensitivity, 90% positive predictive value and 86% negative predictive value. Nine patients in the active toxocariasis group had an AI value lower than 32%, while in the chronic toxocariasis group only one patient had an AI value lower than 32%. Patient 11 in the active group had an AI equal to 67.9% (Table 1); the diagnosis of toxocariasis was established via the immunoblotting detection of local synthesis of anti-*Toxocara* IgG antibodies in both cerebrospinal fluid (CSF) and aqueous humour. To eliminate a possible interference from a high titre of IgM, the AI was retested after inactivation of IgM antibodies via reduction with 2-mercaptoethanol reagent [11]. The AI value was then 58.7%, remaining above the proposed threshold of 32%. The >42% threshold was used to exclude active infection with 92.8% specificity, 68.8% sensitivity, 95.6% positive predictive value and 56.5% negative predictive value. In our adult population, we found that 50% of the patients had high IgG avidity. AI values between 32 and 42% were, thus, considered equivocal. Taking into account the active and chronic groups together, 32% of patients showed equivocal avidity index scores.

**Figure 3:**
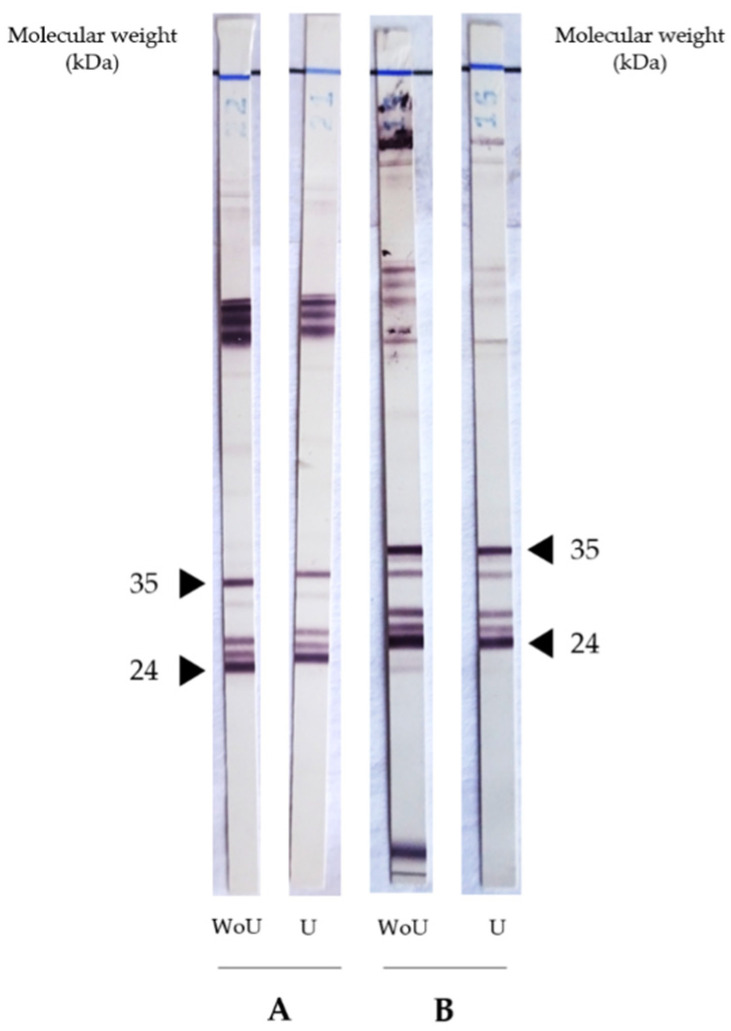
Western blot analysis of sera using the “*Toxocara* Western blot IgG” kit (LDBIO Diagnostics, Lyon, France), which established the presence of *Toxocara* spp.-specific antigens between 24 and 35 kDa. These two proteins bands are indicated by the black arrow. (**A**): Active toxocariasis (patient 2); (**B**): chronic toxocariasis (patient 16); U: with urea treatment; WoU: without urea treatment.

We then tested 11 samples from the active toxocariasis group and four samples from the chronic toxocariasis group using Western blot IgG assays with and without pre-treatment of the sample with urea. The Western blot IgG assays are often performed to evaluate the complete disappearance or lower intensity of low molecular-specific bands (24–35 kDa) observed after treatment with urea according to whether or not an active or chronic infection exists. We observed strictly identical profiles for the Western blot assays performed with and without urea for all patients in both groups (Figure 3).

## 3. Discussion

Currently, the diagnosis of human toxocariasis depends primarily on the detection of specific antibodies in the serum. The best serodiagnostic option is using the IgG TES-ELISA as a screening test with confirmation by TES-WB [12]; however, antibody levels cannot be used to differentiate between an active infection and chronic or past infections. Serological avidity tests are known to discriminate between acute and chronic infections, mainly for parasites and viruses, such as *Toxoplasma gondii* and CMV [7,8]. In this context, we hypothesised that an avidity index (AI) could help to exclude an active or recent infection with *Toxocara* spp. An AI is measured by comparing the value of the OD obtained with a conventional ELISA test with the OD obtained after urea treatment; thus, unstable antigen-antibody liaisons (recent infections) will be dissociated, while very strong liaisons (old infections) will not be affected. In our study, we suspected that AI scores could help to confirm or rule out the active and clinically significant phase of the *Toxocara* infection, which could help to decide whether to introduce or abandon antiparasitic treatment.

We employed the NovaLisa ELISA kit (NovaTec Immunodiagnostica GmbH, Dietzenbach, Germany), which is currently used in our routine diagnostics to detect specific anti-*Toxocara* IgG directed against the synthetic *Toxocara canis* antigen. Considering the significant cross-reactions observed in patients infected with other nematodes of the genera *Ascaris*, *Anisakis* and *Ancylostoma*, we excluded patients who were positive for other helminthiasis infections (*Strongyloides* spp., *Trichinella* spp., *Schistosoma* spp., *Taenia solium*, *Echinococcus granulosus* and *Fasciola hepatica*). According to the manufacturer, the sensibility and specificity of this ELISA assay kit are greater than 95%. All sera tested in our study were confirmed by TES-WB. This involved 46 patients divided into two groups, namely “active toxocariasis” (n = 14) and “chronic toxocariasis” (n = 32) groups. According to the AI scores obtained for both the chronic and active toxocariasis groups, we were able to propose two thresholds: first, an AI score lower than 32% is suggestive of active infection; secondly, a threshold higher than 42% excludes an active infection and rules out the involvement of *Toxocara* in the present clinical manifestation. In our study, nine out of 14 patients in the “active toxocariasis” group had anti-*Toxocara* IgG antibodies with an AI score lower than 32% and no patients in the “chronic toxocariasis” group, except patient 45, had an AI score lower than 32%. Obviously, our cohort included a limited number of patients, especially in the “active toxocariasis” group, and it would be interesting to prospectively evaluate this 32% index threshold. Here, five patients had active toxocariasis with AI scores greater than 32%, including patients 10, 12 and 14, with AI scores of 37.44%, 34.25% and 33.73%, respectively, which were fairly close to the threshold and in the equivocal zone. Patient 11 had an AI score of 67.93%, which was significantly higher than the established threshold. This patient, who was aged 81, presented with sudden deafness and bilateral granulomatous pan-uveitis associated with meningitis symptoms. Anti-*Toxocara* antibodies were found in both his aqueous humour and CSF samples, with no associated eosinophilia. Symptoms resolved after treatment with albendazole and corticosteroids. It could, therefore, be that the high specific IgG avidity in this patient was related to the incubation time of the disease. It is known that the incubation period for toxocariasis can range from a few months to a few years for ocular toxocariasis [8]. Interestingly, in our study, all patients with uveitis had an AI higher than 30% (Figure 2). Regarding patient 10, she presented with three recurrent episodes of abdominal pain over the preceding seven months associated with hypereosinophilia, which improved after treatment with albendazole. Her AI score of 37.44%, which was close to the threshold, was achieved during the third episode. We can, therefore, hypothesise that this patient, who owned dogs and cats, regularly contaminated herself. Various studies have correlated the measure of antibody avidity to the serological responses to vaccination [13,14,15,16]. It is clear that the quality of the humoural response is, at least partially, linked to the avidity of antibodies. In some cases measuring avidity can help to assess the quality of protection induced by vaccination, or in our case, by natural infection. Rudzińska et al. demonstrated that low and medium levels of avidity persisted in subsequent examinations over 2–3 years in children suspected of *Toxocara* infection [17]. The low AI score in our patient may have explained the recurrent infections. Antiparasitic treatments, which the patient received twice, may also have played a role in slowing down the maturation of antibodies, as demonstrated for the *Toxoplasma* IgG avidity, which is influenced by macrolide treatment [18]. It would also be interesting to test the evolution of AI during recurrent *Toxocara* symptomatic infections in furthers studies. Within the “chronic toxocariasis” group, one patient (patient 45) had an AI lower than 32%. This patient presented with encephalitis within the context of atypical fibromyalgia without eosinophilia. The lumbar puncture showed no evidence of local IgG anti-*Toxocara* synthesis in CSF. In conclusion, for this patient who had an AI lower than 32%, there was insufficient evidence to implicate *Toxocara* in their clinical manifestations; however, we did discover a history of travel to India five months prior. Although the prevalence of *T. canis* in India is not well known, the country is a highly endemic area for ascariasis; it is known that patients harbouring *Ascaris lumbricoides* have a positive antibody response to *T. canis* ES antigen [19]. *A. lumbricoides* and *T. canis* infections can remain asymptomatic, and the low AI score observed in patient 43 may hypothetically reveal a muted infection by either of these parasites. Few studies have focused on toxocariasis avidity assays, and all of them have only studied the prevalence of low avidity levels in a given population, without linking them to the clinical setting [17,20,21,22]. All of these studies used the ‘bind and break’ method with urea as the chaotropic agent. Rudzińska et al. and Boldis et al. used the only marketed assay, whereas Dzieman et al., as in our study, used an in-house avidity test [17,21,22]. The major drawback is that there is no method for unambiguous confirmation of *Toxocara* infection, so diagnosis is based on clinical, epidemiologic and laboratory findings (eosinophilia and increased total IgE). In these studies, the sensibility of low AI scores to confirm active toxocariasis ranges between 22.4 and 43.8%. In our study, all of the patients’ medical records were reviewing one-by-one to classify patients as having active or chronic toxocariasis, before testing their AI. Considering the threshold of 32% to confirm an active infection, the sensitivity and specificity of the IgG avidity assay were 64.3% and 96.9%, respectively. Considering the threshold >42% to exclude an active infection, the sensitivity and specificity of the IgG avidity assay were 68.8% and 92.8%, respectively. Dziemian et al. showed that 94·2% of positive sera collected from patients reporting infection >6 months ago had high IgG avidity values, confirming distant toxocariasis, using an in-house avidity assay with a threshold of 50%. Boldis et al. showed sensitivity and specificity values of 43.8% and 83.3%, respectively, for a manufactured avidity assay with a threshold of 40% [22]. Furthermore, the occurrence of low-avidity IgG antibodies in the eosinophilic group (42.1%) was significantly higher than in the non-eosinophilic group (22.0%). In our study, we did not find this same trend. These data should be supplemented by testing other similar ELISA kits based on TES antigens or other antigens to conduct an in-house avidity assay. For example, the antigen chosen as the target for a specific IgG may also influence the AI. This was observed for the rubella virus, since the avidity of the anti-glycoprotein membrane E1 IgG increased in strength two years after infection, while the avidity of anti-glycoprotein membrane E2 and anti-protein C (capsid) IgG remained weak and stable [23]. Avidity maturation also occurs differently depending on the immunoglobulin subclass [24,25]. For example, hydatid patients showed enhanced production of low-avidity anticarbohydrate IgG2, as well as high-avidity antipeptide IgG4 antibodies [25]. Some studies supplement the ELISA avidity test with an avidity immunoblot analysis of the reaction intensity and antigenic band identification, such as in the diagnosis of Q Fever and strongyloidiasis for example [9,10]. In our study and in the literature, the *Toxocara* IgG avidity assay showed much poorer sensitivity and specificity levels than those validated for toxoplasmosis [26]. As such, this assay should be accompanied by other immunological markers. Indeed, immunoblot assays can help to identify major antigens responsible for affinity maturation. The *Toxocara* Western blot IgG technique uses low-molecular weight recombinant antigens (24–35 kDa) that are specific to the genus *Toxocara*. In our study, immunoblot assays were not able to discriminate between active and chronic infection, while the comparative immunological profiles with and without urea treatment were strictly identical. We are aware that the number of tested subjects with active toxocariasis was too small to draw definitive conclusions. At present, this parameter seems to serve better for confirmation of active toxocariasis infections rather than for exclusion of recently acquired infections. Further studies on the evolution of the anti-*Toxocara* antibody response due to various anti-parasitic treatments, the status of the patient’s immunity, the recurrence of infections and time need to be performed to better understand this neglected disease.

## 4. Materials and Methods

### 4.1. Population Study

This retrospective cohort study was conducted at the Parasitology-Mycology Laboratory of IHU Méditerranée Infection (Marseille, France). We included sera from patients with a positive *Toxocara* ELISA test confirmed by a positive immunoblot assay between January 2017 and November 2019. Patients with positive serology for other helminths were excluded. A total of 46 sera samples from 46 patients were obtained. Medically relevant information such as demographic characteristics, clinical presentation (long-term abdominal pain, asthmatic cough, dyspnoea, hepatomegaly, itchy rash or pruritus, ocular and neurological symptoms), laboratory abnormalities (eosinophilia, IgE titre) and the possible existence of a previous positive *Toxocara* serology, medical treatments, additional findings by imaging techniques and the final medical report were collected for each patient. On the basis of this information, patients were classified into two groups: active toxocariasis (n = 14) and chronic toxocariasis (n = 32) (Table 1). The average age was 62 and the sex ratio (M/F) was 1.72.

### 4.2. Serology and Avidity Measurements

Serology screening with ELISA IgG immunoassay and serology confirmation were performed using a NovaLisa kit (NovaTec Immunodiagnostica GmbH, Dietzenbach, Germany) and *Toxocara* Western blot LDBIO kit (LDBIO Diagnostic, Lyon, France) according to the manufacturer’s instructions.

**Figure 4:**
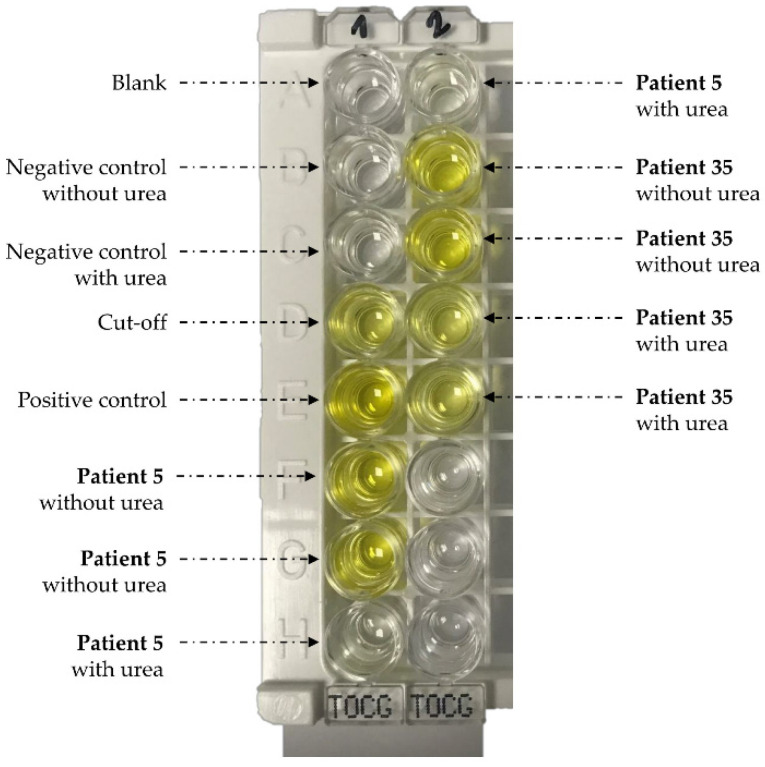
Avidity measurements using the NovaLisa kit (NovaTec Immunodiagnostica GmbH, Dietzenbach, Germany). Patient 5 and patient 35 corresponded to active toxocariasis with low avidity and chronic toxocariasis with high avidity, respectively.

The NovaLisa kit allows qualitative immunoenzymatic determination of antibodies against *Toxocara canis* based on the ELISA technique (Figure 4). Microtitre strip wells are pre-coated with synthetic *T. canis* antigens to bind to corresponding antibodies of the sera. After washing the wells to remove all unbound sample materials, horseradish peroxidase (HRP)-labelled protein A conjugate is added. This conjugate binds to the captured *T. canis* specific antibodies. The immune complex formed by the bound conjugate is visualised by adding tetramethylbenzidine (TMB) substrate. The intensity of this product is proportional to the amount of *T. canis*-specific antibodies in the sera. Absorbance at 450 nm is read using an ELISA microwell plate reader (Labsystems Multiskan RC^®^). Samples are considered positive if the absorbance value is higher than 10% over the cut-off (i.e., > 11 NTU, see below). Results in NovaTec Units (NTU) are calculated as follows:

([Patient absorbance value × 10])/(Cut-off absorbance value) = x NTU

To measure the *Toxocara* IgG avidity index, we used a modified protocol by adding an incubation step with a commercial reagent containing urea (Vidas^®^ CMV IgG avidity II, BioMérieux Marcy-l’Etoile France). Urea is a protein-denaturing reagent that dissociates the antibody-antigen complex with weak affinity [27]. It is commonly used to measure IgG avidity in toxoplasmosis and CMV infections. The avidity assay was performed on all sera with an IgG titre greater than or equal to 11 NTU and according to the technical specifications of the NovaLisa kit, except for two additional steps. First, the IgG titres were adjusted to 15 NTU for all samples, using the diluent reagent included in the NovaLisa kit. If an IgG titre was between 11 and 15 NTU, the sample was tested undiluted. Secondly, the urea treatment was performed after the incubation of sera and before to dispense the conjugate. Here, 200 µL of urea solution (6 M) remained in contact with the antibody-antigen complex on the microwell for 15 min at 37 °C. Then, prior to adding the *Toxocara canis* Protein A conjugate, three washing steps were performed for 10 min in the washing solution included in the kit. For untreated samples, urea was replaced by the sample diluent. The avidity index (AI) was calculated by the ratio of the IgG titre NTU of dissociating reagent-treated and untreated samples multiplied by 100. All samples with and without urea treatment were tested in duplicate. Negative and positive reference sera were included in each plate as controls (Figure 4).

### 4.3. Urea’s Effect on Toxocara Western Blot IgG

Urea treatment was subsequently tested on certain sera classified into the chronic or active toxocariasis group (n = 4 and n = 11 respectively) using *Toxocara* Western blot IgG (LDBIO Diagnostics, Lyon, France). All steps were performed according to the manufacturer’s instructions, with the exception of the urea denaturation step. Urea (Vidas^®^ CMV IgG avidity II, BioMérieux Marcy-l’Etoile France) was applied for 15 min to the nitrocellulose strip at room temperature and with constant agitation after the sera incubation step. A10 min three-wash step was performed with the washing buffer provided in the *Toxocara* Western blot IgG kit to remove urea and potentially denatured low-avidity antibodies. The assay continued from the conjugate step according to instructions. Regarding the ELISA avidity assay, we conducted experiments with and without urea in parallel. Profiles for untreated and treated sera were interpreted by two independent operators who were focused on the five specific bands between 24 and 35 kDa.

### 4.4. Statistical Analyses

From the included sera, two optimal thresholds were determined. The first threshold allowed active toxocariasis to be excluded for the patients with chronic toxocariasis. The specificity was defined as the ratio of the number of avidity results lower than the threshold for active toxocariasis versus the number of active toxocariasis cases. The sensitivity was defined by the ratio of the number of avidity results higher than the threshold for chronic toxocariasis versus the number of chronic toxocariasis cases. The second threshold allowed confirmation of active toxocariasis. The specificity was defined as the ratio of the number sera samples with an avidity result higher than the threshold for chronic toxocariasis versus the number of chronic toxocariasis cases. The sensitivity was defined by the ratio of the number of avidity results lower than the threshold for active toxocariasis versus the number of active toxocariasis cases. Positive predictive values and negative predictive values were assessed.

## 5. Conclusions

IgG avidity assays have been developed for several infectious diseases, although there has been little research focused on toxocariasis diagnosis. The AI could offer supplementary information and could be included in decision-making algorithms comprising clinical, eosinophilia and IgE level data and imaging testing; however, this assay still requires standardisation of the method and further studies in larger populations before it will be useful in routine clinical practice.

## Figures and Tables

**Table 1:** Patients’ medically relevant data and toxocariasis classifications.

Patient	Gender	Age	NovaLisa (NTU) ^1^	Avidity Index (%)	Clinical Group	Eosinophilia	IgE (kUI/L) ^2^	Clinical Presentation	Complementary Information	Treatment		Evolution of Symptoms	Final Diagnosis
										ABZ	IVE		
1	M	61	22.05	18.58 *	Active	N	-	Diffuse myalgia associated with chronic pruritus and abdominal pain		Y	Y	Favourable (except pruritus = pruritus sine materia)	Toxocariasis
2	F	19	19.57	20.97 *	Active	Y	-	Long-term abdominal pain associated with a major hyper eosinophilia		Y	Y	Favourable for eosinophilia	Toxocariasis
3	M	77	36.43	21.45 *	Active	Y	918	Itchy rash and pruritus	Previous serum negative (2 years before)	Y	Y	Favourable	Toxocariasis
4	M	53	29.74	22.78 *	Active	N	-	Acute confusional and cerebellar syndrome evolving for 5 days	Presence of anti-*Toxocara* antibodies in the CSF	ND	N	Favourable	Toxocariasis
5	M	56	35.37	22.79 *	Active	Y	25.1	Bipulmonary transplant patient—respiratory degradation	Previous serum negative (2 months before)	N	N	Stable	Toxocariasis
6	M	75	31.42	26.75 *	Active	Y	33,974	Transient eosinophilia associated with persistent neuropathy		Y	N	Favourable for eosinophilia	Toxocariasis
7	F	76	29.47	28.59 *	Active	N	-	Itchy rash and pruritus		N	Y	Favourable	Toxocariasis
8	F	50	19.43	30.81 *	Active	N	-	Granulomatous anterior uveitis manifested by decreased visual acuity in the left eye for 3 weeks		ND	ND	ND	Toxocariasis
9	M	40	53.00	31.47 *	Active	Y	10.9	Eczematiform dermatosis with eosinophilia		N	N	Favourable	Toxocariasis
10	F	60	30.90	37.44 *	Active	Y	591	Long-term abdominal pain		Y	Y	Favourable	Toxocariasis
11	M	81	35.93	67.93 *	Active	N	-	Bilateral granulomatous panuveitis and meningitis manifested by sudden deafness and decreased visual acuity	Presence of anti-*Toxocara* antibodies in both CSF and AH	Y	N	Favourable	Neuromeningeal toxocariasis
12	M	46	17.59	34.25	Active	Y	-	Fever and episodes of urticaria and chills in a patient with psoriasis		Y	N	Persistence	Toxocariasis
13	M	65	39.18	41.68	Active	Y	14.7	Eosinophilic pneumonia		Y	N	Favourable	Toxocariasis
14	F	28	34.69	33.73	Active	Y	>1000	Etiological assessment of eosinophilia—asthma exacerbation and scan images suggestive of larva migrans syndrome		Y	Y	Favourable	Toxocariasis
15	M	48	27.92	32.73 *	Chronic	N	-	Bilateral granulomatous panuveitis		N	N	Favourable	Neurosyphilis with ocular involvement
16	F	33	25.16	33.16*	Chronic	N	-	Persistent headache with recent onset of left hemiparesis	Absence of anti-*Toxocara* antibodies in the CSF	N	N	Persistence	Inflammatory encephalitis of undetermined etiology
17	F	62	23.24	34.76 *	Chronic	N	-	Significant leptomeningitis associated with frontal ischemic stroke		N	N	Recurrent	Probable dysimmune meningoencephalitis
18	F	68	12.84	36.21 *	Chronic	N	-	Bilateral uveitis		N	N	Recurrent	Sarcoidosis
19	M	38	58.00	37.91	Chronic	N	-	Chronic pruritus evolving over 3 years		N	N	Persistence	*Trichophyton mentagrophytes* dermatophytosis
20	M	77	25.17	39.78	Chronic	N	-	Sub-diaphragmatic lymph nodes		N	N	Follow-up in haematology oncology	Low-grade B lymphoma
21	F	69	16.53	39.87	Chronic	N	89.3	Chronic urticaria evolving over 4 years		Y	N	Persistence	Primary hyperparathyroidism
22	M	67	14.86	41.05	Chronic	N	151	Pruritic papular eruptions evolving over 10 years		N	N	Persistence	Cutaneous mastocytosis
23	M	62	28.32	41.53	Chronic	Y	-	Pruritic and erythematous rash of the face, skull and neck	Previous serum positive (2 years before)	N	N	Persistence	ND
24	F	71	13.46	44.42	Chronic	N	-	Chronic urticaria evolving over 1 year		Y	N	Persistence	Hypothesis of a drug-related cause
25	F	74	39.44	44.56	Chronic	N	-	Chronic pruritus		N	Y	ND	Bullous pre-pemphigoid
26	M	70	14.14	46.23	Chronic	N	-	Granulomatous uveitis of the left eye		N	N	Persistence	Neurolyme with ocular involvement (*Borrelia burgdorferi*)
27	M	73	12.68	47.94	Chronic	N	-	Investigation of a pachymeningitis manifested by chronic headaches for more than 10 years and recent episode of visual haze		N	N	Favourable	Hypotension of the CSF in a patient with an implanted spinal cord neurostimulation system
28	F	66	36.97	50.68	Chronic	N	2577	Assessment of exudative ascites associated with cirrhosis		N	N	Persistence	Refractory ascites in the context of child cirrhosis B9
29	F	38	17.96	55.43	Chronic	N	-	Pruritus *sine materia* evolving over 5 months		Y	N	ND	ND
30	M	61	13.10	57.67	Chronic	Y	7666	Erythrodermic psoriasis with chronic eosinophilia		Y	N	Persistence	Eczema in a patient treated for psoriasis
31	M	76	21.26	58.65	Chronic	N	-	Urticaria evolving over several months	Previous positive serum (1 year before)	N	N	Persistence	ND
32	F	57	16.56	58.65	Chronic	N	-	Epigastric pain revealing intra-abdominal adenomegaly		N	N	Favourable	Mesenteric panniculitis
33	M	85	77.59	59.45	Chronic	Y		Erythematous skin lesions in bullous pemphigoid associated with eczema and dermatitis evolving for 2 years	Previous positive (serum 2 years before)	Y	N	Favourable	Diffuse eczematiform dermatosis
34	F	74	11.10	59.84	Chronic	N	28.9	Etiological assessment of pruritus *sine* *materia* for 3 months (limbs)		N	N	Favourable	Dolichocolon
35	M	53	20.52	60.53	Chronic	NR	-	Repetitive uveitis	Previous positive serum (24 months before)	Y	N	Persistence	Axial and peripheral spondyloarthropathy
36	M	75	12.86	61.73	Chronic	Y	-	Chronic dermatitis evolving over one month		N	N	Persistence	Vascularitis
37	M	56	12.24	62.70	Chronic	N	-	Acute anterior uveitis		N	N	Favourable	Probable HSV uveitis
38	M	54	11.32	64.18	Chronic	N	-	Exploration of neurological and psychiatric disorders	Absence of anti-*Toxocara* antibodies in the CSF	Y	N	Worsening	Dysimmune encephalitis
39	M	64	39.33	64.64	Chronic	N	-	Unilateral granulomatous uveitis		N	N	Favourable	No etiology found
40	M	81	136.36	67.39	Chronic	N	-	Bulbar form of amyotrophic lateral sclerosis		N	N	Worsening	Degenerative motor neuron disease
41	M	80	19.63	70.13	Chronic	N	-	Bilateral endophthalmitis after cataract surgery		N	N	Favourable	Improvement under antibiotics
42	M	63	78.33	71.38	Chronic	Y	-	Asthenia and chronic eosinophilia		Y	Y	ND	ND
43	M	72	12.97	74.07	Chronic	Y	26.8	Discovery of eosinophilia during a follow-up of Minkowski–Chauffard disease		Y	N	Follow-up in haematology–oncology	ND
44	F	74	35.70	87.80	Chronic	Y	-	Chronic cough evolving over more than 40 years associated with eosinophilia		N	N	Follow-up in pneumology	Bronchial hyperreactivity with bronchial dilatation
45	F	71	20.30	23.71	Chronic	N	-	Suspicion of encephalitis in a context of atypical fibromyalgia	Absence of anti-*Toxocara* antibodies in the CSF	N	N	Favourable	Unexplained encephalitis
46	M	40	15.33	33.26	Chronic	Y	1765	Etiological assessment of eosinophilia in an HIV patient		Y	N	Persistence	ND

^1^ Threshold: 11 NTU; ^2^ normal 1–100; * sera tested for urea effect on *Toxocara* Western blot IgG. Y: Yes; N: no; ND: no data; M: male; F: female; ABZ: albendazole; IVE: ivermectine; CSF: cerebrospinal fluid; AH: aqueous humour.

## Data Availability

All data are available within the article.
